# Identification of novel long non-coding RNAs in triple-negative breast cancer

**DOI:** 10.18632/oncotarget.4419

**Published:** 2015-06-10

**Authors:** Xiaokun Shen, Bojian Xie, Zhaosheng Ma, Wenjie Yu, Wenmin Wang, Dong Xu, Xinqiang Yan, Beibei Chen, Longyao Yu, Jicheng Li, Xiaobing Chen, Kan Ding, Feilin Cao

**Affiliations:** ^1^ Department of Surgical Oncology, Taizhou Hospital, Wenzhou Medical University, Taizhou, Zhejiang, China; ^2^ Institute of Cell Biology, Zhejiang University, Hangzhou, China; ^3^ Department of Internal Oncology, Hennan Cancer Hospital, The Affiliated Cancer Hospital of Zhengzhou University, Zhengzhou, Hennan, China; ^4^ Glycobiology and Glycochemistry Lab, Shanghai Institute of Materia Medica, Chinese Academy of Sciences, Shanghai, China

**Keywords:** triple-negative breast carcinomas, long non-coding RNAs

## Abstract

Triple-negative breast carcinomas (TNBC) are characterized by particularly poor outcomes, and there are no established markers significantly associated with prognosis. Long non-coding RNAs (lncRNAs) are subclass of noncoding RNAs that have been recently shown to play critical roles in cancer biology. However, little is known about their mechanistic role in TNBC pathogenesis. In this report, we investigated the expression patterns of lncRNAs from TNBC tissues and matched normal tissues with Agilent Human lncRNA array. We identified 1,758 lncRNAs and 1,254 mRNAs that were differentially expressed (≥ 2-fold change), indicating that many lncRNAs are significantly upregulated or downregulated in TNBC. Among these, XR_250621.1 and NONHSAT125629 were the most upregulated and downregulated lncRNAs respectively. qRT-PCR was employed to validate the microarray analysis findings, and results were consistent with the data from the microarrays. GO and KEGG pathway analysis were applied to explore the potential lncRNAs functions, some pathways including microtubule motor activity and DNA replication were identified in TNBC pathogenesis. Our study revealed that a set of lncRNAs were differentially expressed in TNBC tissues, suggesting that they may play role in TNBC. These results shed light on lncRNAs’ biological functions and provide useful information for exploring potential therapeutic targets for breast cancer.

## INTRODUCTION

Breast cancer is a heterogeneous neoplasm that comprises subtypes with substantial differences in biology and diverse clinical outcomes. As more molecularly targeted therapeutic agents are launched, more clinical remission problems are arising [1]. Therefore, identification of novel therapeutic targets is essential to combat breast cancers, especially those lacking estrogen receptor/progesterone receptor and ErbB2 receptor (triple negative breast cancer, TNBC). TNBC accounts for approximately 10-25% of all breast cancers and is of particular clinical interest due to its tendency to affect younger women and refractory to currently available targeted therapy. The molecular mechanisms for aggressive clinical behavior of TNBC are not fully understood. Various studies show that TNBC have broad and diverse categories for which additional subclasses are needed. Thus, there is considerable interest in understanding potential biomarkers that are significantly associated with TNBC prognosis.

Long non-coding RNAs (lncRNAs) are a subclass of noncoding RNAs (ncRNAs) and have sequence lengths of 200 bp and above [2, 3]. It has become increasingly apparent that lncRNAs contribute to tumor development through many different cellular processes, ranging from transcriptional and post-transcriptional regulation to the control of cell cycle distribution, cell differentiation and epigenetic modifications [4, 5]. LncRNAs modulate gene transcription regulation by rearranging chromatin via chromosomal looping and by affecting the binding of transcription factors. LncRNAs also affect miRNA functions by controlling pre-mRNA splicing or as miRNA sponges. Recently, accumulating evidence indicates that there is aberrant expression of lncRNAs in many cancer types, including glioma, lung, colorectal and hepatocellular cancers, etc [6-8]. Although prognostic lncRNAs expression signatures have been defined for some invasive breast carcinomas, little is known about lncRNAs expression in TNBC, and the underlying pathways regulating TNBC aggressiveness remain poorly understood [9].

Here, we analyzed the expression patterns of lncRNAs and mRNAs in TNBC samples and compared them with the corresponding patterns in adjacent non-tumorous tissue samples. We identified more than 1,200 unique lncRNAs and mRNAs significantly differentially expressed using microarray technology. Several of the differentially expressed lncRNAs were verified by qPCR in other 12 pairs of tissue samples. To determine the biological roles of these differentially expressed lncRNAs and mRNAs, GO and Pathway analyses were used. Coding-non-coding gene co-expression network identified many lncRNAs, such as lncRNA XR_250621.1, that potentially play a key role in TNBC pathogenesis. Our results suggest that lncRNAs expression patterns may provide new molecular biomarkers for the diagnosis of TNBC.

## RESULTS

### LncRNAs and mRNAs expression profiles in TNBC

**Figure 1:**
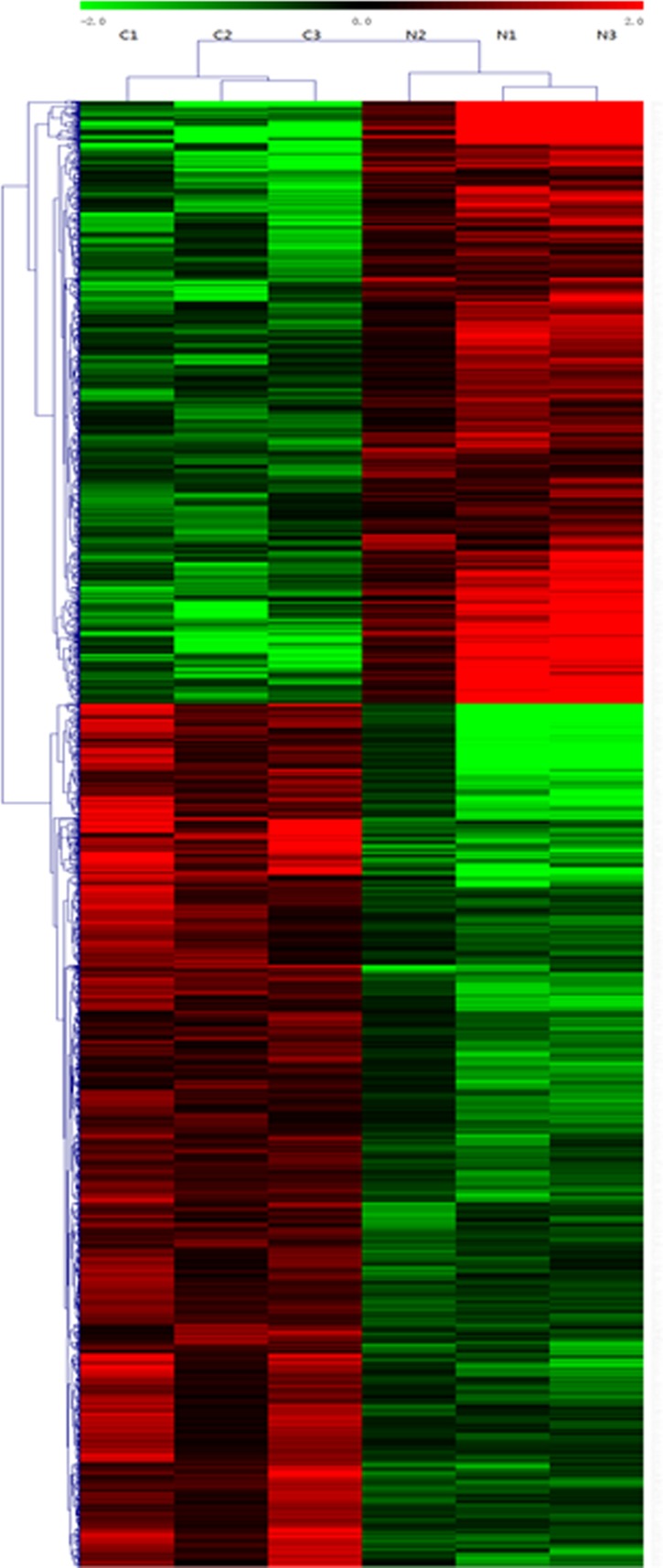
Heat map and hierarchical clustering of lncRNA profile comparison between the TNBC and normal breast samples Each row represents one lncRNA, and each column represents one tissue sample. The relative lncRNA expression is depicted according to the color scale. Red indicates up-regulation; green indicates down regulation. 2.0, 0 and −2.0 are folds changes in the corresponding spectrum, whereas N represents normal breast samples tissue and C represents TNBC tissue. The differentially expressed lncRNAs clearly self-segregated into N and C clusters.

LncRNAs profiling detected 1,403 lncRNAs with significant differential expression levels with at least a two-fold change in TNBC tissues compared with paired normal tissues, with 853 up-regulated and 550 down-regulated respectively. The list of the top 30 differentially expressed lncRNAs identified by microarray analysis was shown in Table 1. Among the dysregulated lncRNAs transcripts, XR_250621.1 (humanseq85285) was the most down-regulated, with an FC of 291.3, whereas NONHSAT125629 (humanseq51739) was the most up-regulated, with an FC of 23.9. Using the same criteria as the lncRNAs, we found 574 up-regulated and 445 down-regulated mRNA transcripts. The most up-regulated and downregulated mRNA transcripts were CDCA2 (NM_152562) and ANKRD30A (NM_052997), with FCs of 22.8 and 155.3, respectively (shown in Table 2). Hierarchical clustering of the lncRNAs and mRNAs profile was performed using cluster 3.0.2; Hierarchical clustering of the expression of the 1,403 lncRNAs and 1,019 mRNAs based on centered Pearson correlation clearly separated TNBC from normal tissues (Figure 1).

**Table 1:** Top 30 aberrantly expressed lncRNAs in microarray for three pairs of TNBC and adjacent non-tumor tissues

Target ID	FC (abs)	p	Regulation	C1	C2	C3	N1	N2	N3	ncRNA_SeqID	Chr
XR_250621.1	291.27	0.03	down	9.31	1.54	3.97	14.14	10.96	14.27	humanseq85285	
NONHSAT012762	164.35	0.04	down	8.00	1.56	1.72	12.20	8.81	12.35	humanseq57970	chr10
TCONS_l2_00002973	144.14	0.04	down	8.09	1.33	2.98	12.59	8.67	12.65	humanseq9097	chr10
NONHSAG005629	135.22	0.04	down	8.22	1.49	3.54	12.67	9.20	12.62	humanseq57206	chr10
NONHSAG050621	133.56	0.01	down	4.09	1.29	2.81	8.88	8.32	12.17	humanseq52435	chr8
NONHSAT012761	128.01	0.04	down	7.94	1.24	3.46	12.45	8.49	12.70	humanseq57969	chr10
NONHSAT127452	113.99	0.01	down	2.81	1.66	1.40	7.76	7.49	11.12	humanseq52434	chr8
XR_252733.1	105.54	0.03	down	7.33	1.50	2.51	11.57	8.26	11.68	humanseq83671	
TCONS_l2_00002976	99.20	0.04	down	8.08	1.55	3.87	12.28	8.59	12.53	humanseq9047	chr10
NONHSAT012773	90.54	0.04	down	6.95	1.74	1.87	11.13	7.47	11.46	humanseq57973	chr10
TCONS_l2_00002971	62.00	0.04	down	7.64	3.46	2.77	11.67	8.00	12.06	humanseq9095	chr10
NONHSAT121750	57.36	0.00	down	2.38	1.45	1.53	7.62	6.19	9.09	humanseq49910	chr7
NR_104061.1	55.50	0.04	down	7.57	3.66	2.70	11.53	8.19	11.59	humanseq86747	10
NONHSAT012774	54.24	0.03	down	6.95	4.22	2.64	11.26	8.11	11.72	humanseq57974	chr10
TCONS_l2_00002977	52.98	0.04	down	5.64	1.44	1.34	9.75	5.82	10.02	humanseq9048	chr10
TCONS_l2_00002974	52.00	0.04	down	7.59	4.23	2.76	11.56	8.29	11.83	humanseq9098	chr10
TCONS_l2_00002972	41.39	0.04	down	6.35	2.56	2.81	10.39	6.79	10.65	humanseq9096	chr10
NR_026916.1	35.88	0.04	down	6.91	2.50	4.67	11.04	7.57	10.97	humanseq88505	
TCONS_l2_00002970	35.17	0.04	down	5.42	1.80	4.24	9.97	6.42	10.49	humanseq9094	chr10
NONHSAT016222	33.04	0.03	down	5.97	2.48	3.51	10.08	6.89	10.12	humanseq58945	chr10
NONHSAT136770	31.09	0.02	down	3.00	2.76	1.23	7.78	5.03	9.07	humanseq55779	chrX
NONHSAT004026	27.10	0.02	down	5.15	2.65	1.85	8.86	6.53	8.55	humanseq30344	chr1
NONHSAG048085	26.13	0.01	down	6.41	5.58	5.77	10.52	9.15	12.22	humanseq49906	chr7
NONHSAT009093	24.41	0.02	down	4.69	6.61	4.89	11.01	8.08	10.93	humanseq32309	chr1
NONHSAT125629	23.95	0.03	up	9.95	6.97	7.45	2.17	5.68	2.77	humanseq51739	chr8
NONHSAT066780	23.54	0.02	down	4.72	4.32	5.21	11.14	7.19	9.59	humanseq77727	chr19
NONHSAT012776	23.09	0.01	down	1.86	1.35	2.54	7.45	4.67	7.21	humanseq57975	chr10
XR_133419.2	22.26	0.01	down	1.35	2.01	1.30	6.48	4.06	7.56	humanseq84668	
NONHSAT121746	21.61	0.03	down	1.30	3.18	2.19	6.98	4.35	8.64	humanseq49907	chr7
NONHSAT098133	20.15	0.00	down	1.46	1.46	1.35	6.44	4.45	6.37	humanseq41971	chr4

**Table 2:** Top 30 aberrantly expressed mRNAs in microarray for three pairs of TNBC and adjacent non-tumor tissues

Probe Name	Target ID	Genbank Accession	FC (abs)	p	Regulation	C1	C2	C3	N1	N2	N3	Chr
A_33_P3368985	ANKRD30A	NM_052997	155.29	0.03	down	8.00	1.60	3.85	12.90	9.32	13.06	chr10
A_23_P8820	FABP4	NM_001442	97.90	0.01	down	2.76	1.39	2.12	7.61	7.36	11.14	chr8
A_23_P161940	SCGB2A2	NM_002411	92.77	0.03	down	2.51	1.48	2.36	11.72	4.99	9.24	chr11
A_23_P218047	KRT5	NM_000424	63.47	0.03	down	9.25	3.97	5.83	12.93	10.68	13.41	chr12
A_23_P12533	ANKRD30A	NM_052997	60.17	0.04	down	6.66	1.79	2.69	10.86	7.00	11.01	chr10
A_33_P3320683			51.36	0.03	down	7.43	2.81	4.11	11.59	8.11	11.70	chr10
A_21_P0010304	ANKRD30A	NM_052997	43.24	0.04	down	6.61	1.50	3.86	10.56	7.24	10.47	chr10
A_23_P111583	CD36	NM_001001547	28.94	0.01	down	6.49	5.52	5.98	10.87	9.35	12.33	chr7
A_23_P127781	SCGB1D1	NM_006552	28.85	0.03	down	1.81	2.28	2.15	9.00	4.21	7.57	chr11
A_24_P273756	TP63	NM_003722	27.89	0.05	down	6.25	1.86	3.92	10.11	6.49	9.83	chr3
A_23_P206920	MYH11	NM_001040114	27.31	0.04	down	7.89	3.40	7.85	11.87	9.87	11.72	chr16
A_24_P70183	MYH11	NM_001040113	26.71	0.05	down	7.85	3.31	7.82	11.80	9.61	11.78	chr16
A_24_P260101	MME	NM_007289	25.46	0.02	down	4.02	3.17	1.37	8.30	5.44	8.83	chr3
A_33_P3319486			24.18	0.01	down	3.11	2.40	1.67	7.13	5.48	8.35	chr7
A_24_P123408	ABLIM3	NM_014945	24.12	0.03	down	6.01	2.24	4.15	9.43	6.99	9.75	chr5
A_23_P385861	CDCA2	NM_152562	22.83	0.03	up	9.83	6.92	7.36	2.58	5.42	2.57	chr8
A_23_P323751	FAM83D	NM_030919	22.31	0.03	up	8.36	5.87	7.33	1.44	5.10	1.59	chr20
A_23_P356684	ANLN	NM_018685	21.49	0.02	up	8.91	7.34	7.56	2.05	5.78	2.70	chr7
A_24_P305050	CD300LG	NM_145273	20.28	0.01	down	3.15	4.37	2.88	8.26	6.14	9.03	chr17
A_23_P403284	OTX1	NM_014562	19.52	0.00	up	8.08	8.74	9.78	3.96	5.42	4.35	chr2
A_23_P45185	FIGF	NM_004469	19.18	0.02	down	1.99	2.55	1.51	6.84	4.25	7.75	chrX
A_23_P77493	TUBB3	NM_006086	18.79	0.03	up	10.62	8.63	6.45	4.57	5.16	3.27	chr16
A_23_P169437	LCN2	NM_005564	18.32	0.01	up	8.88	9.44	9.87	6.25	3.62	5.74	chr9
A_24_P413884	CENPA	NM_001809	17.22	0.02	up	7.19	6.01	6.33	1.32	4.51	1.38	chr2
A_23_P315364	CXCL2	NM_002089	16.18	0.03	down	4.47	3.95	2.05	8.93	5.52	8.07	chr4
A_23_P94422	MELK	NM_014791	16.16	0.04	up	9.81	8.94	9.20	3.86	7.84	4.20	chr9
A_24_P844984	PIGR	NM_002644	16.09	0.02	down	4.57	6.24	4.60	10.07	7.47	9.90	chr1
A_23_P218369	CCL14	NM_032963	16.09	0.01	down	5.07	2.38	4.81	7.36	8.10	8.82	chr17
A_24_P331150	CYP4F22	NM_173483	16.00	0.05	down	2.84	2.41	2.39	6.84	3.94	8.87	chr19
A_23_P81280	BTNL9	NM_152547	15.91	0.02	down	3.23	3.72	4.91	7.64	6.66	9.54	chr5

### Validation of the microarray data using qPCR

**Figure 2:**
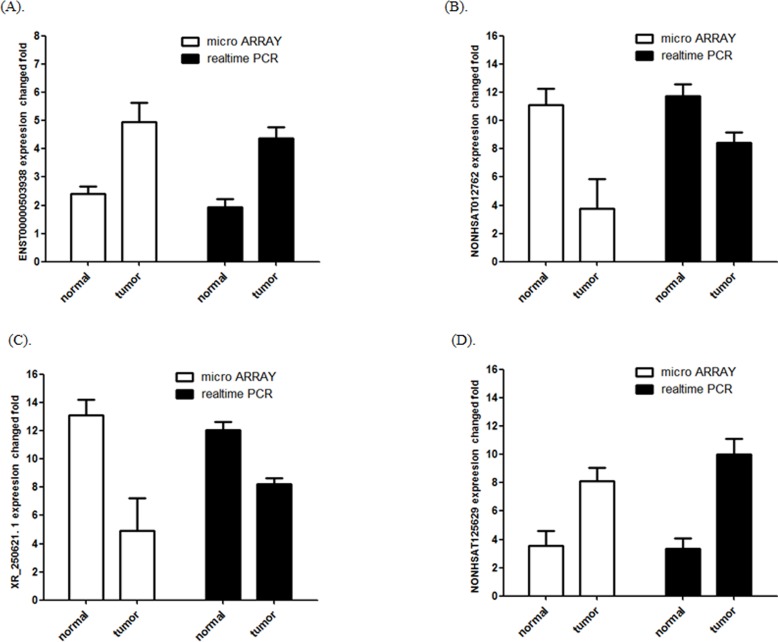
Comparison between microarray data and qPCR results **A.** ENST00000503938, **B.** NONHSAT012762 **C.** XR_250621.1 and **D.** NONHSAT125629 which were determined to be differentially expressed in TNBC samples compared with NT samples in 3 paired patients by microarray was validated by qPCR in 12 paired tissues. The heights of the columns in the chart represent the log-transformed median fold changes in expression across the 12 patients for the lncRNA validation; the bars represent standard errors. The validation results of the lncRNAs indicated that the microarray data correlated well with the qPCR results.

The most upregulated lncRNA XR_250621.1 and downregulated lncRNA NONHSAT125629 were selected for validation using qPCR. In addition, two lncRNAs (ENST00000503938 and NONHSAT012762) were randomly selected to validate the microarray consistency using qPCR. The results demonstrated that lncRNAs NONHSAT125629 and ENST00000503938 were up-regulated and that XR_250621.1 and NONHSAT012762 were down-regulated in the tumor samples compared with NT samples (Figure 2). These qPCR results are consistent with the microarray data.

### Go and KEGG pathway analysis

**Figure 3:**
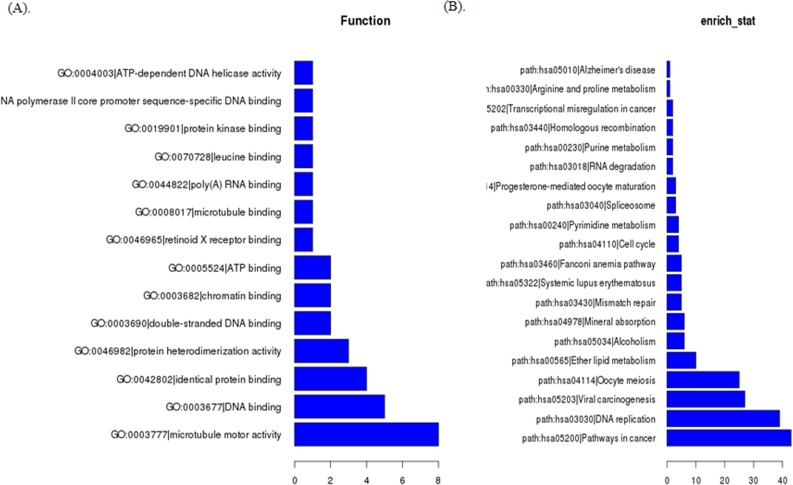
GO analysis **A.** and KEGG Pathway analysis **B.** of aberrantly expressed lncRNAs in TNBC.

To predict the functions of the lncRNAs, we adopted method originally demonstrated in this paper [10]. Briefly, we first calculated the co-expressed mRNAs for each of the differentiated lncRNAs, and then we conducted a functional enrichment analysis of this set of co-expressed mRNAs. The enriched functional terms were used as the predicted functional terms for each given lncRNA. To explore potential biological associations, we ran GO and Pathway analysis with the top 500 differentially expressed lncRNAs and mRNAs. GO analysis indicated that several functional pathways were enriched. Among these pathways, protein binding, fibroblast growth factor-activated receptor activity, structural constituent of ribosome, protein kinase binding and poly(A) RNA binding signaling were the most closely associated with TNBC (Figure 3A). Furthermore, using the same criteria as the GO analysis, KEGG Pathway analysis showed that some pathways corresponded, including ribosome, pathways in cancer, NOD-like receptor signaling pathway, cell cycle, DNA replication, etc. (Figure 3B).

### Construction of co-expression network

**Figure 4:**
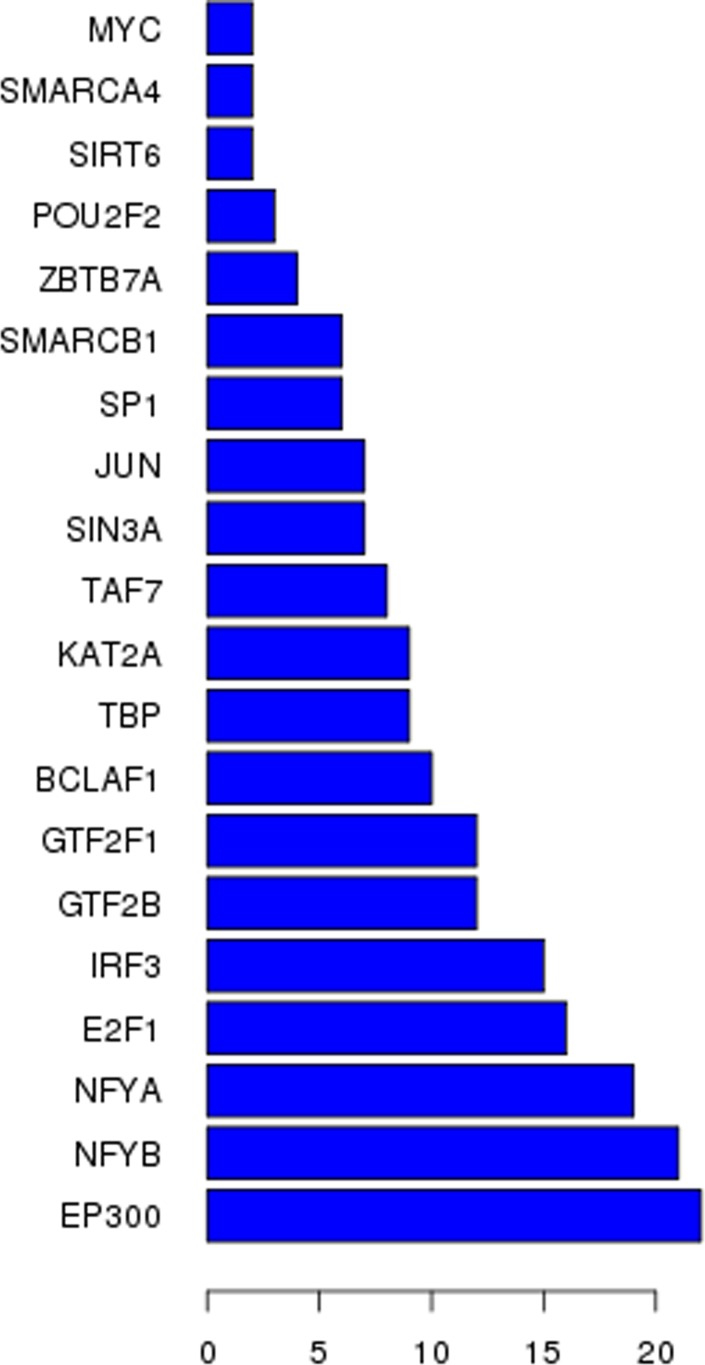
Top transcriptional factors profiling based on aberrantly expressed lncRNAs in TNBC

To explore which lncRNAs and mRNAs play a critical role in TNBC progression, we constructed a co-expression network based on the correlation analysis between the differentially expressed lncRNAs and mRNAs. LncRNAs and mRNAs with Pearson's correlation coefficients of no less than 0.99 were used to construct the network. To explore lncRNAs that possibly have trans-regulating functions, we compared the mRNAs that coexpressed with these lncRNAs with the mRNAs that are regulatory targets of certain Transcription factors (TFs). Results show that EP300, NFYB and E2F1 may play central roles in lncRNAs process (Figure 4). The co-expression network indicated that one mRNA or lncRNA might correlate with one to ten lncRNAs (Figure 2S). The co-expression network may suggest that the inter-regulation of lncRNAs and mRNAs is involved in TNBC.

## DISCUSSION

Pathogenesis of breast cancer remains unclear; therefore, further study of breast cancer is of great importance. As lncRNAs constitute an important class of gene expression regulatory factors, their aberrant expression would inevitably lead to abnormal gene expression levels, which might result in tumorigenesis [2, 5]. To date, there have been few studies studying lncRNAs expression profile in breast cancer or predicating the association of lncRNA expression with clinical pathological features and outcomes in breast cancer [11, 7, 12, 9, 13, 14]. Thus, lncRNAs have opened a new field of breast cancer genomics. Although there are no drugs that act against lncRNAs presently, it will be fascinating to observe whether drugs could be developed that specifically target lncRNAs. Notably, lncRNAs can be detected in human body fluids and hold great promise as biomarkers.

In the present study, we investigated lncRNAs expression signature of TNBC tumor samples from patients. With abundant and varied probes amounting to 78,243 human lncRNAs and 30,215 coding transcripts in the microarray, a large number of lncRNAs could be determined quantitatively and significant differential expression in cancer tissue compared to normal breast tissue was observed. 1,758 lncRNAs and 1,254 mRNAs were found to be significantly differentially expressed. In addition, qRT-PCR was employed to validate the microarray analysis findings, and results were consistent with the data from the microarrays. These results revealed that there were unique lncRNAs expression signatures in these tissues. However, the majority of differentially expressed lncRNAs corresponded to novel transcripts of unknown functions [4, 15]. In order to obtain insight into lncRNA target gene functions, GO analysis and KEGG pathway annotation were applied to the lncRNA target gene pool. GO analysis revealed that the number of genes corresponding to down-regulated mRNAs were larger than that corresponding to up-regulated mRNAs. These pathways may play important roles in the occurrence and development of TNBC. Increased understanding of the role of these potential endogenous lncRNAs in breast cancer cells could provide additional insight on the role these pathways play in mediating breast cancer progression.

An increasing number of studies have demonstrated that a number of lncRNAs are not transcriptional noise, but have important functions, such as regulating gene expression at various molecular levels, including protein, RNA, miRNA and DNA [16, 17]. Few studies have focused on how lncRNA genes themselves were transcriptionally regulated. Yang et al. developed a system by which users could browse transcription factor binding sites in the regulatory regions of lncRNAs [18]. However, lncRNAs are temporally and spatially expressed and regulated, and motif-based sequence analysis cannot capture the dynamic regulation of lncRNAs by transcription factors. In this study, we constructed a transcription factors-lncRNAs-mRNA network based on expressions in the TNBC tissue and binding sites in the regulatory regions of a specific lncRNA. Results showed that EP300, NFYB and E2F1 played central roles in lncRNAs process and TNBC development, which were consist with previous reports [19-21]. As more data become available, it will facilitate the research on the transcriptional regulation of lncRNAs.

Several limitations should be acknowledged for this study. First, gene expression microarray have limited dynamic range and lack the ability to discover novel features as splice isoforms or fusion transcripts. RNA-seq technology promises to unravel previously inaccessible complexities in the transcriptome, such as allele-specific expression and novel promoters and isoforms. However, datasets produced are large and complex and interpretation is not straight forward. Second, the sample size of each dataset is relatively small, the significance and robustness of the signature requires further confirmation, ideally with large prospective patient cohorts with prognostic date. Last but not least, although the roles of the lncRNAs in TNBC pathogenesis are presently unclear, our findings suggest that lncRNAs deserve further studied. Additional functional investigations of these lncRNAs on cancer cell lines and xenograft models may increase our outstanding of their roles in determining TNBC prognosis.

To summarize, comprehensive in-depth analysis of the expression profiles of lncRNAs was executed in this study. A set of lncRNAs with differential expression were found in TNBC compared with normal breast tissue. Furthermore, potential roles for these lncRNAs in the regulation of protein binding, fibroblast growth factor-activated receptor activity, structural constituent of ribosome, protein kinase binding and poly (A) RNA binding signaling pathways will be identified. Further investigation of the lncRNAs identified in this study will likely focus on their biological functions and their association with TNBC. Our study provides useful information for exploring potential therapeutic targets for breast cancer.

## MATERIALS AND METHODS

### Ethics statement

This study was approved by the human ethics committee of the Zhejiang Taizhou Hospital, People's Republic of China. All patients are informed and have declared written informed consent that their samples can be used for research.

All patients received tumor resection at Zhejiang Taizhou Hospital and were diagnosed with TNBC histopathologically after surgery. Immunochemical staining of estrogen receptor/progesterone receptor and ErbB2 receptor in 3 samples are shown in Figure S1. There was no radiotherapy or chemotherapy prior to surgery. 3 paired samples were used for microarray analysis of lncRNAs and 12 paired samples were used for an extra evaluation by real-time PCR. Demographic and clinical characterizations of the study population are summarized in Table S1.

### Tissue collection and RNA extraction

Paired TNBC tissues and adjacent normal breast tissues from every subject were snap-frozen in liquid nitrogen immediately after resection and stored at −80 °C until use. The mirVana^TM^ RNA Isolation Kit (Ambion, Foster City, CA, United States) was used to extract total RNA from frozen samples, according to the manufacturer's protocols, which were then eluted with 100 mL of nuclease-free water. Total RNA was quantified with the NanoDrop ND-2000 (Thermo Scientific) and the RNA integrity was assessed using Agilent Bioanalyzer 2100 (Agilent Technologies).

### LncRNA and mRNA microarray expression profiling

The microarray profiling was conducted in the laboratory of the OE Biotechnology Company in Shanghai, People's Republic of China. The sample labeling, microarray hybridization and washing were performed based on the manufacturer's standard protocols. Briefly, mRNA was purified from total RNA after removal of rRNA by using an mRNA-ONLY Eukaryotic mRNA IsolationKit (Epicentre Biotechnologies, USA). Then, each sample was transcribed to double strand cDNA, then synthesized into cRNA and labeled with Cyanine-3-CTP. The labeled cRNAs were hybridized onto the Human lncRNA array V4.0 (4 × 180 K, Agilent), including the global profiling of 78,243 human lncRNAs and 30,215 coding transcripts. After washing, the arrays were scanned with the Agilent Scanner G2505C (Agilent Technologies). Feature Extraction software (version 10.7.1.1, Agilent Technologies) was used to analyze array images and extract the raw data. Genespring (Version 12.5, Agilent Technologies) was employed to finish the basic analysis of the raw data. To begin with, the raw data were normalized with the quantile algorithm. The probes that had at least 1 condition out of 2 conditions flagged as “P” were chosen for further data analysis. Differentially expressed lncRNAs and mRNAs were then identified through fold-change as well as *P* values calculated with *t*-test. The threshold set for up- and down-regulated genes was fold change ≥ 2.0 and *p* value ≤ 0.05. Afterwards, Hierarchical Clustering was performed to display the distinguishable lncRNAs and mRNAs expression patterns among the samples.

### Functional group analysis

GO analysis and KEGG analysis were applied to determine the biological roles of these differentially expressed mRNAs, base on the latest KEGG (Kyoto Encyclopedia of Genes and Genomes) database (http://www.genome.jp/kegg/). The *p* value (Hypergeometric-*P* value) denotes the significance of the pathway correlated to the conditions. The recommend *p*-value cut-off is 0.05.

### Construction of the co-expression network

Potentially trans-regulated protein-coding genes were defined as coexpressed and beyond 100 kb in genomic distance from, or on the other allele of, differentially expressed lncRNAs. The lncRNAs-Transcription factors (TFs) network was constructed using hypergeometric cumulative distribution function with the help of MATLAB 2012b (The MathWorks). The graph of the lncRNAs-TFs network was drawn with the help of Cytoscape 3.01 (Agilent and IBS). If the intersection of these two groups is large enough (*P* < 0.01, calculated by hypergeometric cumulative distribution function and FDR < 0.01, under the control of the Benjamini and Hochberg procedure), then we predict that these lncRNAs possibly participate in pathways regulated by these TFs. The recently released ENCODE data on TFs and their regulatory targets were used in our analysis

### Real-time quantitative reverse transcription-PCR

A two-step reaction process was used for quantification reverse transcription (RT) and PCR. Each RT reaction consisted of 0.5 μg RNA, 2 μL of Primer Script Buffer, 0.5 μL of oligo dT, 0.5 μL of random 6 mers and 0.5 μL of Primer Script RT Enzyme Mix I (TaKaRa, Japan), in a total volume of 10 μL. Reactions were performed in a GeneAmp^®^ PCR System 7500 (Applied Biosystems) for 15 min at 37 °C, followed by heat inactivation of RT for 5 s at 85 °C. The 10 μL RT reaction mix was then diluted 10-fold in nuclease-free water and held at −20 °C. At the end of the PCR cycles, melting curve analysis was performed to validate the specific generation of the expected PCR product. All experiments were done in triplicate. The expression levels of lncRNAs were normalized to glyceraldehyde-3-phosphate dehydrogenase and were calculated using the 2-ΔΔCt method. The primer sequences were designed in the laboratory based on the DNA sequences and is shown in Table S2.

### Statistical analysis

The Statistical Program for Social Sciences (SPSS) 18.0 software (SPSS, Chicago, IL, United States) was employed to perform all the statistical analyses. All data were expressed as the mean ± SD or proportions where appropriate. For comparisons, paired *t*-tests and unpaired *t-*tests were performed where appropriate. GraphPad Prism 5.0 for Microsoft Windows (GraphPad Software, San Diego, CA, United States) was used to plot all graphs. *P* values of 0.05 (two-tailed) were considered statistically significant.

## SUPPLEMENTARY MATERIAL FIGURES AND TABLES


