# Supplementary material for: Identification of novel long non-coding RNAs in triple-negative breast cancer
Source: Oncotarget. 2015 Jun 10;6(25):21730–9. doi: 10.18632/oncotarget.4419 (PMC4673299; doi:10.18632/oncotarget.4419)
Supplement: Supplementary file 1 [file oncotarget-06-21730-s001.pdf]

Identification of novel long non-coding RNAs in triple-negative breast cancer

Supplementary Material

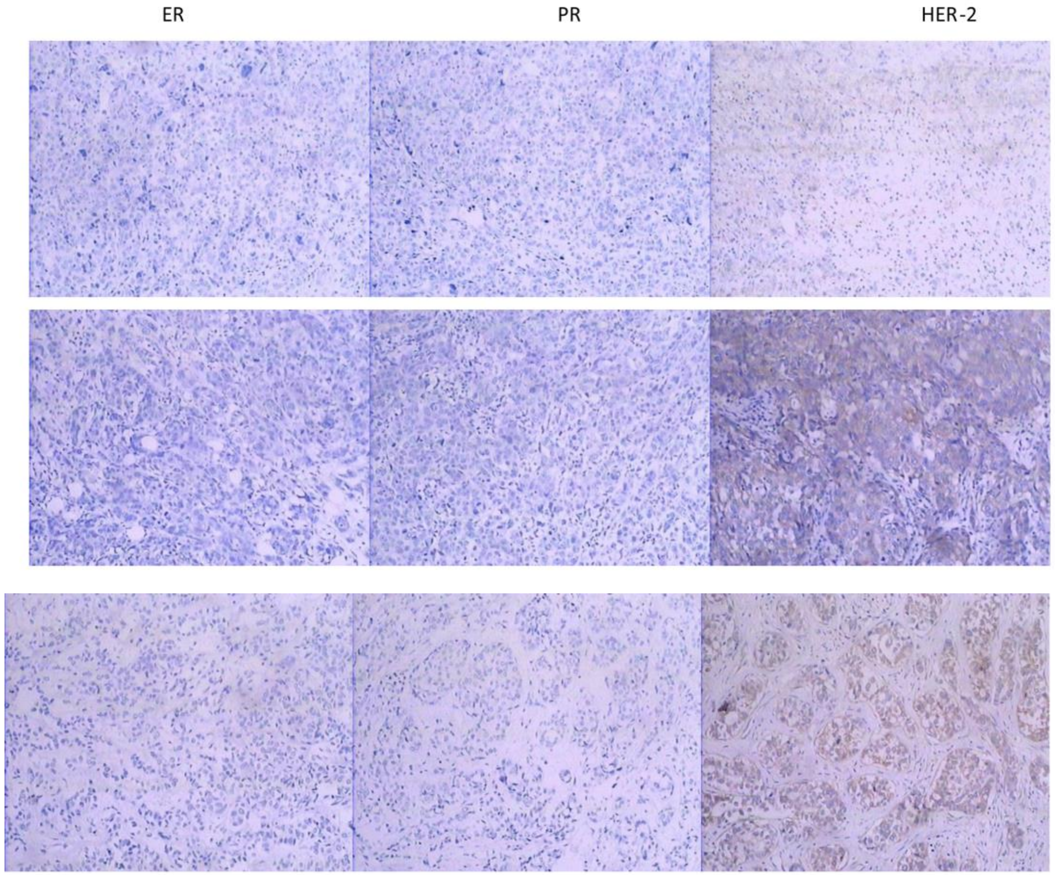

Fig.1S: Immunochemical staining of estrogen receptor/progesterone receptor and ErbB2 receptor of 3 TNBC samples for microarray analysis

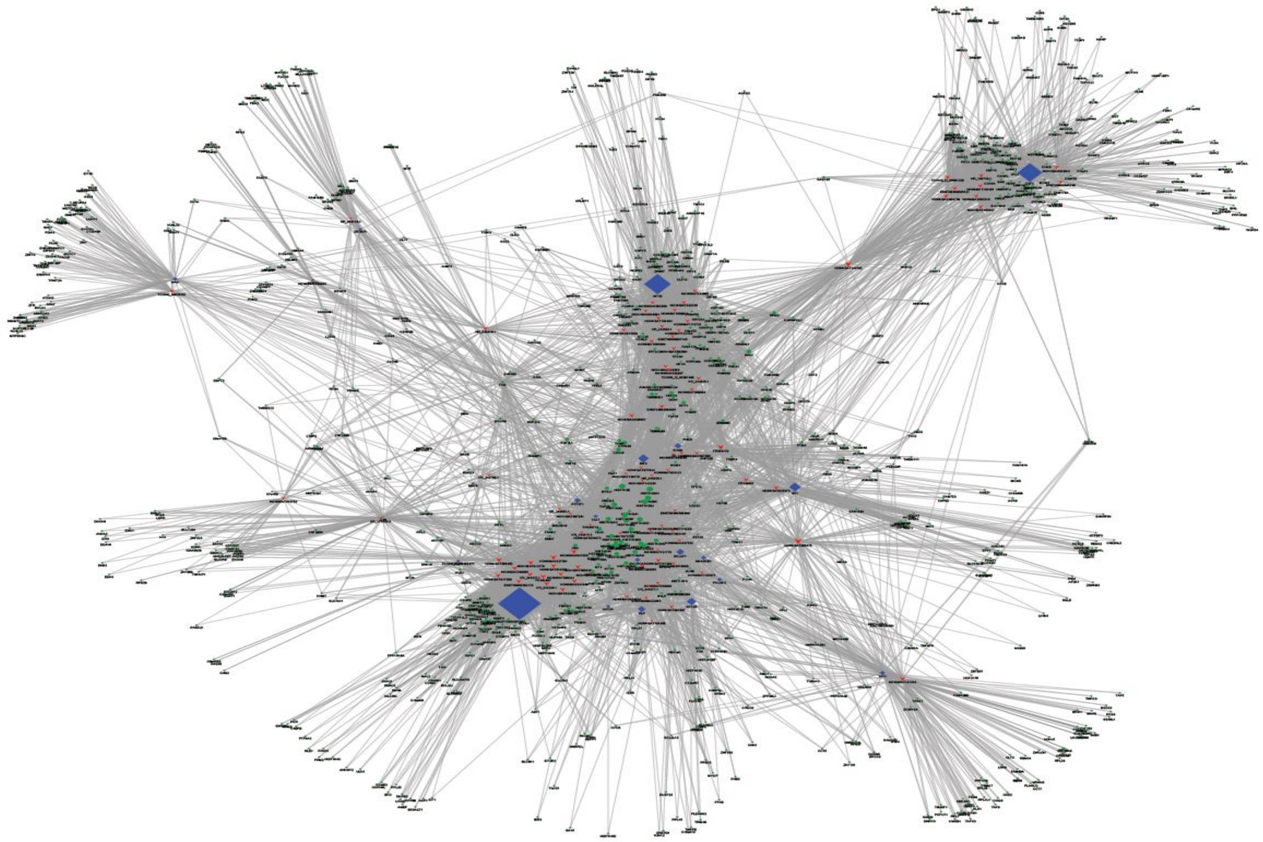

Fig.2S LncRNA-mRNA network was constructed based on the correlation analysis between the differential expressed lncRNAs and mRNAs. In the network, blue nodes represent transcription factors, red nodes represent the lncRNAs and green nodes represent the target mRNAs. The size of node is proportional to the outgoing link number. The thickness of outgoing link represents statistical relationship to the number of occurrences of the results proportionally.

Table S1 The demographic characteristics of 15 TNBC cases

Patient Number	Patient Code	Sex	Age（years）	Diagnosis	Surgery date	ER	PR	HER2
1	WMY	F	50	Breast cancer	2012.07.12	−	−	1−2+
2	ZHX	F	49	Breast cancer	2012.07.11	−	−	2+
3	CYF	F	46	Breast cancer	2012.07.11	−	−	2+
4	FDL	F	59	Breast cancer	2012.07.13	−	−	1−2+
5	FSF	F	41	Breast cancer	2010.06.29	−	−	1−2+
6	HSX	F	59	Breast cancer	2010.08.31	−	−	+
7	JHF	F	58	Breast cancer	2010.06.11	−	−	1−2+
8	HLY	F	56	Breast cancer	2010.03.25	−	−	2+
9	GJQ	F	39	Breast cancer	2010.06.29	−	−	1−2+
10	LCF	F	55	Breast cancer	2011.09.13	−	−	−
11	LMY	F	57	Breast cancer	2012.07.13	−	−	−
12	ZAW	F	54	Breast cancer	2011.07.02	−	−	2+
13	CXX	F	72	Breast cancer	2011.01.05	−	−	+
14	WXN	F	41	Breast cancer	2010.02.08	−	−	+
15	LXX	F	52	Breast cancer	2012.07.13	−	−	+

Table S2 The primer sequences for Real-time PCR.

No.	lncRNAs	Forward primer	Reverse primer	Product length(bp)	Ta(℃)
1	ENST00000503938	TTGTAAAGAGCTGTAAAGGGCA	TCAGCATTTCGGTTACATGAT	104	53
2	NONHSAT012762	GACCAGTCTGGTGTTCTT	GTGCACAGCTCAGGATCA	100	55
3	XR_250621.1	GAATATACGGGCTACAGAGGA	TGTTTGGATTCTGATGGGAG	149	60
4	NONHSAT125629	GAGCCCTTCCGGACAGAG	AGGAGGTAATTCGGCATGCT	187	60
